# Age-Related Changes in Immunological and Physiological Responses Following Pulmonary Challenge

**DOI:** 10.3390/ijms18061294

**Published:** 2017-06-17

**Authors:** Edmund J. Miller, Helena M. Linge

**Affiliations:** 1 The Center for Heart and Lung Research, The Feinstein Institute for Medical Research Manhasset, New York, NY 11030, USA; emiller@northwell.edu; 2 The Elmezzi Graduate School of Molecular Medicine, Manhasset, New York, NY 11030, USA; 3 Hofstra Northwell School of Medicine, Hempstead, New York, NY 11549, USA; 4 Lund University, Faculty of Medicine, Department of Clinical Sciences Lund, 221 00 Lund, Sweden

**Keywords:** aging, pneumonia, remote organ damage, sepsis

## Abstract

This review examines the current status of knowledge of sepsis and pneumonia in the elderly population and how the dynamics of the pulmonary challenge affects outcome and consequences. Led by an unprecedented shift in demographics, where a larger proportion of the population will reach an older age, clinical and experimental research shows that aging is associated with certain pulmonary changes, but it is during infectious insult of the lungs, as in the case of pneumonia, that the age-related differences in responsiveness and endurance become obvious and lead to a worse outcome than in the younger population. This review points to the neutrophil, and the endothelium as important players in understanding age-associated changes in responsiveness to infectious challenge of the lung. It also addresses how the immunological set-point influences injury-repair phases, remote organ damage and how intake of drugs may alter the state of responsiveness in the users. Further, it points out the importance of considering age as a factor in inclusion criteria in clinical trials, in vitro/ex vivo experimental designs and overall interpretation of results.

## 1. The Aging Population and Risk of Pulmonary Infections with Severe Consequences

The current life expectancy in the US is 78.8 years [1] and it is estimated that by the year 2030, 20% of the US population will be older than 65 [2]. Aging is a major risk for the development of lung diseases both acute and chronic [3,4,5]. In healthy individuals, the maximum functional ability of the lungs occurs at 20–25 years of age [6]. In following years, the functions undergo progressive debilitation e.g., decreases in static elastic recoil, compliance of the chest wall, and in the strength of respiratory muscles. The effect of age on responsiveness of the lung, in particular, on innate immunity is apparent even in middle age [7]. The changes in pulmonary physiology, pathology, and function, common in older populations, are associated with altered responses and susceptibility to lung infections [8]. Thus, in later life, susceptibility to lung infections increase, and pneumonia occurs in around 25–44 cases per 1000 of elderly individuals [9], and in those >65 years, pneumonia is the major cause of death from infection [10]. In many respects, the reasons for the altered susceptibility remain unclear.

## 2. Aging and Acute Lung Injury

Understanding age-related changes of the lung and their impact on cardiopulmonary responses during infectious insult for maintenance of homeostasis is of critical importance for developing novel therapeutic strategies for the treatment of the older patient. Acute lung injury (ALI), or acute respiratory distress syndrome (ARDS) as currently defined in humans [11,12], is worldwide a major clinical state affecting approximately 1 in every 1250 individuals annually [13]. Age is a determining factor in the incidence of ARDS affecting 16 per 100,000 person-years in young (15–19 years of age) and 306 per 100,000 person-years in older individuals aged 75–84. Similarly, the associated mortality increases from 24% (at 15–19 years) to 60% (>85 years). Additional studies confirm age as a major indicator of clinical outcome in ALI [14,15], and show that there is a male predominance in the incidence within older individuals [13,16,17,18,19]. Although much effort has been made to develop more appropriate ventilator and fluid management strategies, mortality from ARDS remains high [20,21,22].

Although, ALI has many etiologies, pneumonia and sepsis are leading causes [22,23,24]. Community-acquired pneumonia (CAP) among elderly individuals is often inflicted by bacterial respiratory infections primarily caused by Gram positive pathogens [25]. Gram-negative and viral pneumonias also contribute to the overall pneumonia-related deaths in the elderly population. Pneumonia has great impact on society and the affected individuals and their families by being a primary cause of morbidity, mortality and socioeconomic cost, resulting in >50,000 deaths in the US alone [26].

## 3. Gram Positive Bacteria and Pneumonia in the Elderly Population

A common cause of pneumonia is infection by *Staphylococcus aureus* (*S. aureus*), especially in elderly patients [27] and a complication associated with mechanical ventilation [28]. Advanced age and pulmonary *S. aureus* infection have been shown in critically ill patients as prime risk factors in pneumonia and sepsis [29].

While there are numerous strains of *S. aureus*, expressing a wide variety of exo- and enterotoxins, extracellular enzymes, superantigens etc., with differences in antibiotic resistance, specific host-adapted properties, all strains of *S. aureus* have cell envelopes containing both lipoteichoic acid (LTA) and peptidoglycan (PGN) [30]. These two molecules themselves can have profound effects on immune function and can lead to multiple organ failure, shock and death. While most hospitalized patients with sepsis, or ALI arising from sepsis, may, due to the use of antibiotics, have few live bacteria in the lungs, they have significant amounts of bacterial components within the airspaces [31,32].

## 4. Neutrophils in the Lungs of Elderly Individuals

In the early stages of infection, neutrophils leave the blood stream and enter tissues to clear them from invading pathogens. However, neutrophil functions are altered with age in many different models, as summarized in Table 1 [33]. Collectively, in vitro results examining neutrophil function during aging are somewhat difficult to interpret unambiguously as stimuli and time points vary. Use of particulate matter versus live bacteria to examine phagocytosis, or Gram− vs. Gram+ bacteria impact on reactive oxygen species (ROS) production are examples of this [34]. Tseng et al., suggest that impaired ability to form neutrophil extracellular traps (NETs) in aged mice limits the ability of confinement of a pulmonary infection to the lungs, and allows systemic dissemination and fulminant sepsis [35]. Taken together, aged neutrophils exhibit impairments in their microbicidal ability.

Evidence suggests that neutrophil recruitment and in vivo chemotaxis are dysregulated in the lungs of both elderly patients and models that use older animals. In some infection models neutrophil recruitment is impaired at early time-points, while in others increased numbers of neutrophils accumulate at the site of infection and fail to disperse later, most likely as a result of impaired efferocytosis. Indeed, delayed neutrophil apoptosis exacerbates acute lung injury, and transbilayer (endothelial–epithelial) migration induces significant decreases in apoptosis related caspase (-3, -8 and -9) activities [36]. In addition, the reduced apoptosis is correlated with downregulation apoptosis-related gene expression including Fas ligand and tumor necrosis factor receptor 1 (TNF-R1) expression.

By concomitantly measuring chemotactic chemokines in plasma and the alveolar space following intra-tracheal delivery of LTA/PGN in young and old mice, we observed a steeper chemotactic gradient across the endothelial–epithelial bilayer of the alveolar wall for the older individuals [37]. The lungs of old mice did not contain higher levels of neutrophils at baseline but following stimulation, the amount was significantly elevated in the older mice. We hypothesize that the local chemotactic response is increased in the lungs, but as the plasma levels remained low (or delayed) a steeper chemotactic gradient resulted. Compared to the young individuals, old mice had higher local levels of chemotactic mediators, lower plasma levels and showed a higher rate of neutrophil influx to the lungs, than did the young mice. Similar studies in rats showed results consistent with those in mice: the older animals had an increased chemotactic gradient in favor of the airspaces, which was associated with a greater accumulation of neutrophils and protein [38]. This is mirrored by a study showing that elderly patients with respiratory tract infections caused by *S. pneumoniae*, and aged mice infected with *Pseudomonas aeruginosa* had an increased and prolonged neutrophil accumulation in the lung parenchyma as compared to their young counterparts [39,40,41]. Neutrophil chemotaxis is normally a tightly regulated process. Age-related changes can however result in a delayed clearance of pathogens [42,43]. The dysregulated neutrophil migration may cause accumulation and can contribute to prolonged inflammation and damage of the pulmonary tissue [44]. Neutrophil dysfunction plays an important role in the inability of older individuals to mount an effective and timely orchestrated response to bacterial pathogens, and is likely to impair resolution of pulmonary inflammation.

## 5. The Endothelium as Stage for Events Influencing Development and Outcome of Infection

Acute lung injury (ALI) causes a great morbidity and mortality in critically ill patients [45]. ALI is associated with dysfunction in the pulmonary endothelial barrier [46]. It is characterized by severe hypoxemia with acute onset and bilateral infiltrates in patients not suffering from heart failure or fluid excess [47]. A compromised endothelial barrier is of vital importance for pulmonary integrity. Concurrent events including oxidative stress, responses to inflammatory mediators, and uneven regulation of proliferatory and apoptotic pathways induce loss of endothelial function [47,48]. Taken together the events occurring in or in close proximity to the pulmonary endothelium result in leakage of fluid and protein into interstitial spaces, edema formation, multiple organ inflammation, and ultimately multiple organ injury, remote from the lung [49,50,51]. Angiopoietins (Ang-) act on cell junctions to modulate endothelial permeability and Ang-2 in particular blocks junction stabilization. Elevated angiopoeitin-2 increases have been associated with fluid overload, remote organ damage, and death from septic shock in humans and believed to contribute to vascular leakage [52]. In a paper describing how the structure of gut vessels changes, as animals age it was noted that Ang-2 was elevated [53]. The in vivo reduction of Ang-2 release by inhibition of increased oxygen saturation and improved pulse distention in mice following pulmonary challenge with *S. aureus* cell wall components [54]. In addition, in vitro findings using pulmonary vessels show that production of Ang-2 is regulated by two distinct mechanisms, constitutive and bacterial agent-stimulated [55]. Determination of whether either of these pathways could be used for therapeutic targeting to reduce Ang-2 secretion, and ultimately protect the pulmonary endothelial barrier function, and may be beneficial to prevent the microvascular and hemodynamic instabilities associated with ARDS during pneumonia and sepsis. Such targeting could be of extraordinary benefit to the aging population. Ex vivo experiments where explanted primary microvascular endothelial cells from young (3 months) and old (24 months) rats were treated with sera obtained from sepsis patients and healthy controls, revealed an increased sensitivity in the “old” cells to oxidative stress and cell injury as measured by death receptor activation, and apoptotic cell death (caspase 8 activity and caspase 3 activity, respectively) [56]. Although the experimental design of that study does not clarify which factor(s) in the septic sera is responsible, it does show that explanted primary microvascular endothelial cells retain their age-affected responsiveness.

The clinical situation does not normally lend itself to prevention of the endothelial barrier dysfunction. It is only after the injury has occurred that the detrimental effects are evident. Thus the therapeutic window occurs following the injury, when repair of the barrier function is critical. Resolution and repair of the injured endothelium and reestablishment of normal endothelial barrier functions are crucial for recovery of organ function. However, the in vivo mechanisms regulating the transition from barrier injury to repair and regulating endothelial barrier recovery remain to be fully elucidated. The severity of injury will impact the extent of repair, and also affect the process and dynamics of transitioning from injury to repair [47]. Thus, it is important that future studies address both the injury and repair phases and determine the key age-associated differences that lead to poor outcome in the older individual. It will likely also influence the long-term survival and quality of life after a septic event with pulmonary engagement.

## 6. Cellular Senescence

Cellular senescence is a stress-induced, controlled state of cell cycle arrest which occurs in all stages of the lifespan [57]. It is marked by a series of possible upregulated markers and pathways (ARF, p16^INK4a^, p38MAPK, NF-κB, β-galactosidase, and shortening of telomere length). Its heterogeneity and the lack of a single acknowledged marker has long kept the research field from developing a complete understanding of its development and consequences. In aging, senescent cells accumulate which leads to functional impairments. This has led to a new term, homeostenosis, being coined to mark the signature of senescence on the organism level [58]. The senescent cell remains a structural component of the tissue but does not respond to the microenvironment (e.g., growth factors or apoptotic stimuli), as would functional counterparts. There is evidence indicating that senescent cells may be cleared by means of physiological events but the exact circumstances remain unknown [57]. The delicate micromilieu of the lung, as demonstrated during infection by endothelial permeability, intra-alveolar epithelial and macrophage responses, mounting of inflammatory response, pathogen destruction and, finally, resolution are normally well orchestrated events. Cellular senescence is believed to indicate molecular, rather than chronological age [57]. In this regard it adds the same nuance to cellular biology as frailty does to the holistic evaluation of an individual’s health status. Cellular senescence has similarities with fibrosis, particularly in the lungs [59]. Elegant work on idiopathic pulmonary fibrosis has recently shown that senescence markers are present within the lungs of this patient group and, interestingly depleting the senescent cells restored pulmonary health in an animal model [60]. Depletion of senescent cells as an early intervention may offer a novel opportunity for treating pulmonary fibrosis. What the consequences of depletion of such cells would have on fighting pulmonary infection constitutes an interesting avenue for future research.

## 7. The Quest for Biomarkers in Sepsis—Are the Findings also Relevant for Use in the Elderly Population?

In recent years, much work has focused on identifying one or a panel of biomarkers which would predict organ failure or outcome during the septic event. In understanding the dynamics of the clinical course of sepsis, a state of high diversity and clinical heterogeneity [61], it is important to examine the relevance of proposed markers also in the elderly population. Procalcitonin (PCT) has been considered a promising candidate, and assessment of this plasma marker together with interleukin (IL)-10, -6 and -5 in an emergency room admittance study were useful in predicting intensive care unit (ICU) admissions but held no value in predicting death of sepsis in the elderly population [62]. More assuring, the sepsis-related organ failure assessment (SOFA)-scores and the abbreviated mortality emergency department sepsis (abbMEDS) scores remained useful in predicting both ICU admissions and deaths. Global proteomic approaches e.g., mass spectrometry analyses of sepsis patient sera and neutrophil derived proteins may offer new insights in understanding subgroups of patients [63,64]. In the era of precision medicine, the raster of age adds another level of complexity to an already complex problem. It remains important for novel technologies and approaches to consider age as a factor in the inclusion criteria in clinical trials, in vitro/ex vivo experimental designs and overall interpretation of results.

## 8. Long Term Effects Following Pneumonia or Sepsis

A retrospective study of 50,119 males (mean age of 77.9 years) found an increased incidence of cardiovascular events within a three-month period of hospital admission for community-acquired pneumonia (CAP) [65]. A Taiwanese study found an increase in cardiovascular events in the long term follow-up after sepsis in patients (*n* = 66,961) with chronic kidney disease [66]. In our mouse model we observed age-dependent differences in both renal [67] and cardiac responses during the pulmonary challenge [37]. Examining the proteasome function in heart muscle following pulmonary challenge revealed that proteasome function was significantly reduced in the old mice, compared to young counter parts. In severe sepsis, cardiac function is often compromised [24], and patients with cardiac dysfunction during sepsis have a worse prognosis than those without [68]. Studies of long-term follow up after a septic event including other etiologies than pneumonia show that quality of life, morbidity and mortality are affected [69,70].

Sepsis survivors show an increased use of health care as measured by hospital readmissions, increased morbidity and long term increased mortality. The outcome in sepsis survivors is traditionally evaluated 30 or 90 days post discharge. In a large American study of 3315 Medicare participating hospitals, 30% of sepsis patients who received treatment between 2008 and 2011 for sepsis were readmitted to hospital within 30 days of discharge [71]. Similarly, 40% of ARDS survivors had one or more hospitalizations in the first year following discharge [72]. 5-year mortality in sepsis survivors was close to 60% in one Norwegian study [73], and 87% in extra corporeal membrane oxygenation (ECMO) treated patients admitted for sepsis or respiratory failure [74]. Lower health related quality of life, mental or physical impairments or inability to perform work is reported in several studies [75,76,77]. Survivors also had a raised tolerance to thermal stimulation indicating damage to small peripheral nerve fibers [76]. Animal studies using mice and *Drosophila melanogaster* as models of survival after sepsis, show shortening of telomere length [78], cognitive impairments, intermittent failure to recognize novel objects [79], disrupted insulin signaling in the brain, reduction of glucose stores [80], altered metabolic pathways [81], and sustained inflammation [79,80]. Due to the complexity of the findings, and uncertainties of causality, it remains difficult to draw any conclusions of how age alone may influence survival. As pointed out by others [82,83], other factors come into play, like comorbidities, frailty, nutritional status and other predisposing factors. The area of long term survival after sepsis is likely to expand when treatments improve and the number of survivors increase. The future may show which treatments, pathogen- or host specific factors lead to the symptoms reported by survivors and what may be done to limit these adverse effects in both young and old individuals.

## 9. Factors influencing long term outcomes after pneumonia and sepsis

Statins are a group of therapeutics used to lower the plasma level of low-density lipoprotein (LDL) cholesterol. In humans, drug intake and its influence on development and outcome of pneumonia (CAP), show that 30-day mortality rate was reduced in patients taking statins, and that statin intake could reduce the risk of CAP in diabetic patients [84,85]. Statins did not influence survival in a mouse model of sepsis but survivors had a lower clinical score [86]. Interestingly, the authors of that study found results indicating a role for statins in reversing microvascular dysfunction and limiting neurological sepsis-induced damages, which are known to lead to cognitive impairment. Surprisingly, statins have also been shown to have an effect on the aging process of the human lung, by attenuating the decline in forced expiratory volume in 1 secondFEV1 and forced vital capacity (FVC) that is normally seen with aging [87]. Whether this effect is different from, or associated with the effects seen following infectious insult (e.g., pneumonia) remain to be determined. The example of statin intake demonstrates that studies of responsiveness to infection in the elderly population are likely to be affected by a number of confounding factors, including body composition and life-time dietary choices. A recent study showed that the microbiota of the gut, in combination with the increased permeability of the same during aging, influences the low grade inflammation associated with aging, so-called “inflamm-aging” [88]. The researchers could show that microbiota-transplantation into germ-free mice (18–22 months of age, which normally do not exhibit inflamm-aging) caused them to accrue a similar inflammation profile as their wild type counterparts. The potential molecular links between statins, microbiota, gut permeability, induction of cellular senescence, and its potential targeting for therapeutic gain, provides a new landscape of research opportunities for understanding and altering inflammatory set-points that hitherto have been associated with aging.

## 10. Summary

Bacterial pulmonary insults cause endothelial injury and disrupt endothelial barrier integrity, resulting in fluid and protein leakage into interstitial spaces and edema formation. However, even in this most severe form of acute lung injury the gaseous exchange function of the lung can normally be supported, and the majority of deaths are attributed to sepsis or multiple organ failure rather than the failure of respiration. This suggests that the age associated changes in immunological and physiological responses following pulmonary challenge have more important ramifications than dysregulation of gaseous exchange alone. Thus, a long term goal of future studies should be to understand the role of the lung on organ dysfunction, particularly why the lung dysfunction can have such profound effects on hepatic and renal dysfunction, and blood pressure, so that we can define specific therapeutic targets that will improve both survival from pneumonia, especially in the elderly, and improve the quality of life of survivors.

## Figures and Tables

**Table 1:** Ageing effects on important neutrophil functions (courtesy of [33]).

Function	Species
Cytokine production	Human
ROS generation	Human, mouse, rat
Chemotaxis	Human
Ability to form NETs	Human, mouse
Degranulation	Human

ROS *=* reactive oxygen species; NETs = neutrophil extracellular traps.
